# Power and sample size calculations for testing the ratio of reproductive values in phylogenetic samples

**DOI:** 10.1093/aje/kwae378

**Published:** 2024-10-10

**Authors:** Lucy D’Agostino McGowan, Shirlee Wohl, Justin Lessler

**Affiliations:** Department of Statistical Sciences, Wake Forest University, Winston-Salem, NC 27109, United States; Department of Immunology and Microbiology, The Scripps Research Institute, La Jolla, CA 92037, United States; Department of Epidemiology, Johns Hopkins Bloomberg School of Public Health, Baltimore, MD 21205, United States; Department of Epidemiology, Johns Hopkins Bloomberg School of Public Health, Baltimore, MD 21205, United States; Department of Epidemiology, The University of North Carolina at Chapel Hill, Chapel Hill, NC 27599, United States; Carolina Population Center, The University of North Carolina at Chapel Hill, Chapel Hill, NC 27599, United States

**Keywords:** sample size, power, pathogen sequence data, phylogenetic samples

## Abstract

The quality of the inferences we make from pathogen sequence data is determined by the number and composition of pathogen sequences that make up the sample used to drive that inference. However, there remains limited guidance on how to best structure and power studies when the end goal is phylogenetic inference. One question that we can attempt to answer with molecular data is whether some people are more likely to transmit a pathogen than others. Here we present an estimator to quantify differential transmission, as measured by the ratio of reproductive numbers between people with different characteristics, using transmission pairs linked by molecular data, along with a sample size calculation for this estimator. We also provide extensions to our method to correct for imperfect identification of transmission-linked pairs, overdispersion in the transmission process, and group imbalance. We validate this method via simulation and provide tools to implement it in an R package, phylosamp.

## Introduction

Analysis of pathogen sequence data has revolutionized how we study infectious disease, helping us address a myriad of questions ranging from identifying the geographic origin and expansion of pathogens,^1^ to reconstructing outbreaks,^2^^,^^3^ to estimating the transmissibility of emerging infectious diseases.^4-6^ While many of the methods used to answer these questions are quite sophisticated and leverage core principles of evolutionary and epidemic theory,^7^^,^^8^ much work is based on simply identifying transmission-linked pairs.^2^^,^^9^ Regardless of the sophistication of the methods used, the quality of the inferences we make from pathogen sequence data is determined by the size and structure of the sample of pathogen sequences used to drive that inference. However, there remains limited guidance on how to best structure and power studies when the end goal is phylogenetic inference.

In previous work, we showed how sample size (i.e., the number of sequences considered) and other study characteristics influenced the identification of transmission-linked pairs, including how many pairs a study could expect to identify and the number of “false positives” (i.e., people deemed to be transmission linked that were not) that would occur if the criteria used to define transmission linkage is imperfect. However, while this work provided guidance on study design to identify transmission-linked pairs, it did not explore whether the resulting set of pairs identified would be sufficient to answer an overarching inferential question.

Here, we attempt to take that next step for one key question we might ask from pathogen sequence data: are some people more likely to transmit a pathogen than others? Specifically, we present an estimator for the ratio of reproductive values between groups (i.e., individuals with different characteristics) along with a sample size calculation for this estimator. We explore the impact of imperfect observation of transmission-linked pairs (i.e., the sensitivity and specificity of the linkage criteria) on required sample size, and we address how other aspects of the transmission process, such as super spreading and differential susceptibility, impact this estimate.

## Methods

### Defining transmission pairs

Often we are interested in asking whether there is differential transmissibility of a pathogen between people with different levels of some covariate, $G$. For example, are older people more like to transmit than younger people? A core measure of differences in transmissibility is the ratio of the reproductive number of those with covariate level $A$ ($G=A$), denoted $R$*_A_*, versus those with covariate level *B* ($G=B$) , denoted $R$*_B_*. We assume the characteristic of interest does not influence who you will be infected by or who you will infect; however, it may influence *how many* people you will infect (i.e., transmission is not differential based upon the relationship between the value of *G* of the infector and infectee). For example, while we may be exploring whether men transmit more than women, we are assuming that men are no more likely to infect other men than women, and vice versa.

### Definitions and distributional assumptions

We denote the size of the total infected population as *N* with $\pi$_A_ representing the proportion for whom $G=A$ and $\pi$_B_ = 1 − $\pi$_A_. Hence, *N_A_* = *N*$\pi$*_A_* is the total infected population for whom $G=A$, and *N_B_* = *N*$\pi$*_B_* is the total infected population in which $G=B$. Each individual in the infected population can be thought of as a *node* connected to both their infector and their infectees via *edges*. Let us assume that our data consist of pathogen sequences obtained from a simple random sample of infected individuals such that the sampling proportion is $\rho$ and the total number of nodes sampled with each covariate level is as follows: ${M}_A={N}_A\rho$, ${M}_B={N}_B\rho$.

A sampled transmission pair is defined as 2 individuals *i* and *j* who are both included in the sample and are connected by an edge (i.e., *i* infected *j* ). If ${e}_{i\cdot }$ is the total number of infections in the sample caused by infector *i*, then the total number of directed edges in a sample of size *M* is *E*:


\begin{equation*} E=\sum \limits_{i=\mathrm{1}}^M{e}_{i\cdot }. \end{equation*}

The probability that a transmission partner for infector $i$ is included in the sample is $\frac{M-1}{N-1}$ (we subtract 1 from the numerator and denominator to account for the infector) where $M$ is the total number of nodes in the sample. We assume the total number infected by $i$ (${e}_{i\cdot }$) is Poisson distributed with a rate $\lambda =\frac{M-1}{N-1}{R}_{g_i}$^10^ where ${R}_{g_i}$ is the total number of infectees for a given sampled infection $i$ belonging to group $G=g$. Assuming ${e}_{i\cdot }$ is Poisson distributed, the sum of all ${e}_{i\cdot }$ ($E$) is also Poisson distributed with the rate, $M\times \frac{M-1}{N-1}{R}_{pop}$ where ${R}_{pop}$ is the average reproductive number for the infected population (i.e., ${R}_{pop}$ is the total number of transmissions divided by the total number of infected individuals; see Appendix S1 for further intuition).

If we could perfectly observe transmission events, we would be able to observe the frequency of four types of directed edges in our sample:

1.
${E}_{AA}$ : The total number of directed edges in a sample where an individual in group *A* infects another individual in group *A*.
2.
${E}_{AB}$ : The total number of directed edges in a sample where an individual in group *A* infects an individual in group *B*.
3.
${E}_{BA}$ : The total number of directed edges in a sample where an individual in group *B* infects an individual in group *A*.
4.
${E}_{BB}$ : The total number of directed edges in a sample where an individual in group *B* infects another individual in group *B*.

**Figure 1:**
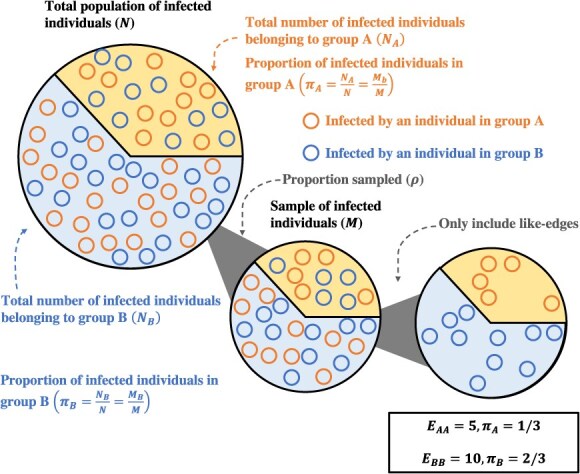
Sampling schema. This diagram begins with an illustration of a total infected population on the left. Each infected individual is denoted as a circle with the background color indicating the group the individual belongs to, orange for group A and blue for group B. The color of the circle indicates who infected that individual. Moving to the right, we first see a simple random of this total population with probability $\rho$ (in this case 0.5). Finally, we see only the like-edges remaining in the final circle on the far right, where “like-edges” mean that the infector and infectee belong to the same group. These edges are what are used in the estimators in the remainder of the methods discussed here.

If these transmission pairs are identified using phylogenetic linkage criteria,^10^ such as the genetic distance between infecting viruses, we often will not know who is the infector and who is the infectee absent additional epidemiologic information (e.g., the timing of symptom onset) or deep sequencing data.^11^ Hence we do not know the directionality of edges and cannot differentiate ${E}_{AB}$ from ${E}_{BA}$ without further assumptions; therefore, we do not know the group of the infector for such edges. However, for concordant edges in a sample, ${E}_{gg}$ for $g\in \left\{A,B\right\}$, we do know the group of the infector; hence, such edges contain information about the group’s relative likelihood of transmitting the pathogen. See Figure 1 for a visual representation of the process described previously.

### Defining an estimator for the ratio of reproductive numbers

Using the definitions and distributional assumptions previously described, we defined an estimator for differential transmission as characterized by the ratio of reproductive numbers between groups and defined our hypothesis test in relation to this estimator.

We assumed that the total number of observable group *g* with concordant edges in a sample of size *M* (edges where both the infector and infectee are included in the sample) is Poisson distributed, with a mean of ${\lambda}_g$, ${E}_{gg}\sim Pois\left({\lambda}_g\right)$ where


(1) \begin{equation*} {\lambda}_g=\frac{M(M-\mathrm{1}\mathrm{)}{R}_g{\pi}_g^2}{(N-\mathrm{1}\mathrm{)}} \end{equation*}

for $g\in \left\{A,B\right\}$. See Appendix S1 for the derivation.

We are interested in testing whether the ratio of *R* values in each group is equal to 1 vs >1; in other words, we conducted the following 1-sided hypothesis test:

${H}_0:{R}_A={R}_B$ against ${H}_1:{R}_B/{R}_A>\mathrm{1}$.

Equivalently, we tested the null hypothesis that $\log \big({R}_B/{R}_A\mathrm{\big)=0}$. Therefore, we can develop a test statistic similar to that specified in Ng and Tang.^12^

Note that the sample estimate for *R_g_* is


(2) \begin{equation*} {\widehat{R}}_g=\frac{E_{gg}(N-\mathrm{1}\mathrm{)}}{M(M-\mathrm{1}\mathrm{)}{\widehat{\pi}_g}^2},g\in \big\{A,B\mathrm{\big\},} \end{equation*}

where ${\widehat{\pi}}_g$ is a known quantity, the proportion that belongs to group *g* in the sample and that is ${\widehat{\pi}}_g={M}_g/M$. Hence, $\frac{{\widehat{R}}_B}{{\widehat{R}}_A}$ is


(3) \begin{equation*} \frac{{\widehat{R}}_B}{{\widehat{R}}_A}=\frac{E_{BB}{\widehat{\pi}}_A^2}{E_{AA}{\widehat{\pi}}_B^2}. \end{equation*}

This gives us the following test statistic:


(4) \begin{equation*} U=\log \big({\widehat{R}}_B/{\widehat{R}}_A\big)=\log \left({E}_{BB}/{E}_{AA}\right)-\log \big({d}^2\mathrm{\big),} \end{equation*}

where $d={\widehat{\pi}}_B/{\widehat{\pi}}_A$. In practice, $d={M}_B/{M}_A$ since ${\widehat{\pi}}_g={M}_g/M$ for $g\in \left\{A,B\right\}$. Applying the delta method,


(5) \begin{equation*} \mathit{\operatorname{var}}(U)={\sigma}_U^2=\frac{\big(N-\mathrm{1}\mathrm{\big)}}{R_A{\pi}_A^2M\big(M-\mathrm{1}\mathrm{\big)}}+\frac{\big(N-\mathrm{1}\mathrm{\big)}}{R_B{\pi}_B^2M\big(M-\mathrm{1}\mathrm{\big)}}. \end{equation*}

This can be estimated by


(6) \begin{equation*} {s}_U^2=\frac{\big(N-\mathrm{1}\mathrm{\big)}}{{\widetilde{R}}_A{\pi}_A^2M\big(M-\mathrm{1}\mathrm{\big)}}+\frac{\big(N-\mathrm{1}\mathrm{\big)}}{{\widetilde{R}}_B{\pi}_B^2M\big(M-\mathrm{1}\mathrm{\big)}}, \end{equation*}

where ${\widetilde{R}}_g$ is any reasonable estimate for ${R}_g$ for $g\in \left\{A,B\right\}$. By letting ${\widetilde{R}}_g={\widehat{R}}_g$ for $g\in \left\{A,B\right\}$, we can use $U/{s}_U$ to test the null hypothesis, resulting in the following statistic,


(7) \begin{equation*} W=\frac{\log \left({E}_{BB}/{E}_{AA}\right)-\log \left({d}^2\right)}{\sqrt{1/{E}_{AA}+1/{E}_{BB}}}. \end{equation*}

Under the null hypothesis, *W* is asymptotically normally distributed, with a mean of 0 and a standard deviation of 1; thus we reject the null hypothesis when $W>{z}_{1-\alpha }$ where ${z}_{1-\alpha }$ is the $100\times \mathrm{(1}-\alpha )$th percentile of the standard normal distribution. Note that this statistic does not exist when *E_gg_* = 0. Thus both groups *A* and *B* would need to have at least 1 set of concordant edges for this to be estimable.

### Power and sample size

Now that we have an estimator, we can calculate the sample size needed to detect prespecified effects with a given power.

We can represent the variance $(N-\mathrm{1}\mathrm{)/}{R}_A{\pi}_A^2M(M-\mathrm{1}\mathrm{)}+(N-\mathrm{1}\mathrm{)/}{R}_B{\pi}_B^2M(M-\mathrm{1}\mathrm{)}$ as $\left[1+\frac{R_A}{d^2{R}_B}\right]\frac{(N-\mathrm{1}\mathrm{)}}{R_A{\pi}_A^2M(M-\mathrm{1}\mathrm{)}}$ where $d={\pi}_B/{\pi}_A$. Under ${H}_1:{R}_B/{R}_A>\mathrm{1}$, we know asymptotically $\log \left({E}_{BB}/{E}_{AA}\right)-\log ({d}^2)$ follows a normal distribution, as expressed here:


(9) \begin{equation*} \log \left({E}_{BB}/{E}_{AA}\right)\!-\!\log \left({d}^2\right)\!\sim\! N\left(\log ({R}_B/{R}_A\mathrm{),}\left[1\!+\!\frac{R_A}{d^2{R}_B}\right]\frac{(N-\mathrm{1}\mathrm{)}}{R_A{\pi}_A^2M(M\!-\!\mathrm{1}\mathrm{)}}\right) \end{equation*}

Therefore the power can be expressed as


(8) \begin{equation*} P=\mathrm{1}-\varPhi \left[{z}_{1-\alpha }-\frac{\log \left(\frac{R_B}{R_A}\right)}{\sqrt{\left[1+\frac{R_A}{d^2{R}_B}\right]\frac{(N-\mathrm{1}\mathrm{)}}{R_A{\pi}_A^2M(M-\mathrm{1}\mathrm{)}}}}\right]. \end{equation*}

Hence, the total sample size needed to achieve $1-\beta$ power under ${H}_1:{R}_B/{R}_A>\mathrm{1}$ is


(9) \begin{equation*} M=\frac{1}{2}\left[1+\sqrt{4\left[1+\frac{R_A}{R_B{d}^2}\right]\frac{\big({z}_{1-\alpha }+{z}_{1-\beta }{\big)}^2}{\log \big({R}_B/{R}_A{\big)}^2}\frac{\big(N-\mathrm{1}\mathrm{\big)}}{R_A{\pi}_A^2}+1}\right], \end{equation*}

where ${z}_{1-\beta }$ is the $\mathrm{100}\mathrm{\big(1}-\beta \big)$th percentile of the standard normal distribution.

### Adjusting for sensitivity and specificity of linkage criteria

When identifying transmission pairs using some phylogenetic criteria, or a combination of epidemiologic and phylogenetic criteria, the transmission pairs actually identified in our sample will be dependent on the sensitivity and specificity of that criteria.^10^ That is, since any criteria for identifying transmission pairs will be imperfect, we will sometimes misclassify pairs of individuals as being part of a transmission pair when they are not (and vice versa). This matters because the expected number of observed transmission pairs (edges) under misclassification, ${E}_{gg}^{\ast }$, differs from the expected number of sampled edges in the absence of misclassification, ${E}_{gg}$. The expected number of observed edges under misclassification is


\begin{equation*} {E}_{gg}^{\ast }={E}_{gg}\big(\eta +\gamma +\mathrm{1}\mathrm{\big)}+\frac{M_g\big({M}_g-\mathrm{1}\mathrm{\big)}}{2}\mathrm{\big(1}-\gamma \mathrm{\big),}g\in \big\{A,B\big\} \end{equation*}

where $\eta$ is the assumed sensitivity level and $\gamma$ is the assumed specificity level.

Therefore, accounting for imperfect sensitivity and specificity, the sample estimate for *R_g_* is as follows,


\begin{equation*} {\widehat{R}}_g=\frac{E_{gg}^{\ast }-\frac{M_g\big({M}_g-\mathrm{1}\mathrm{\big)}}{2}\mathrm{\big(1}-\gamma \big)}{\eta +\gamma -1}\times \frac{\big(N-\mathrm{1}\mathrm{\big)}}{M\big(M-\mathrm{1}\mathrm{\big)}{\widehat{\pi}}_g^2},g\in \big\{A,B\mathrm{\big\}.} \end{equation*}

First, let’s consider the case where specificity is 100%, and we have imperfect sensitivity. Here, the number of edges observed will be reduced from it true value, but out estimator will still be unbiased; hence we can adjust our sample size estimate as follows,


(10) \begin{equation*} M=\frac{1}{2}\left[1+\sqrt{4\left[1+\frac{R_A}{R_B{d}^2}\right]\frac{\big({z}_{1-\alpha }+{z}_{1-\beta }{\big)}^2}{\log \big({R}_B/{R}_A{\big)}^2}\frac{N-1}{R_A\eta{\pi}_A^2}+1}\right], \end{equation*}

where $\eta$ is the estimated sensitivity. However, if specificity is less than 100%, then our observed estimate of ${R}_B/{R}_A$ will be biased towards the null (assuming errors are nondifferential). This bias will further decrease our power. If ${\pi}_A={\pi}_B$, then the following will be a conservative estimate for the sample size needed to achieve a given power


(11) \begin{equation*} M=\frac{1}{2}\left[1+\sqrt{4\left[1+\frac{R_A^{\ast }}{R_B^{\ast }{d}^2}\right]\frac{\big({z}_{1-\alpha }+{z}_{1-\beta }{\big)}^2}{\log \big({R}_B^{\ast }/{R}_A^{\ast }{\big)}^2}\frac{N-1}{R_A^{\ast }{\pi}_A^2}+1}\right], \end{equation*}

where ${R}_A^{\ast }={R}_A\left(\eta +\gamma -1\right)+\left(1-\gamma \right)\left(N-1\right)/2$, ${R}_B^{\ast }={R}_B\left(\eta +\gamma -1\right)+\left(1-\gamma \right)\left(N-1\right)/2$, and $\eta$is the assumed sensitivity level, and $\gamma$ is the assumed specificity level. Alternatively, if a more accurate estimate is required, or ${\pi}_B<{\pi}_A$, then the appropriate sample size can be calculated by solving the transcendental equation that occurs when you replace ${R}_g^{\ast }$ with ${R}_g^{\ast }={R}_g\times \big(\eta +\gamma -\mathrm{1}\mathrm{\big)}+\frac{\big(\big(M{\pi}_g-\mathrm{1}\mathrm{\big)/2\big)}\times \mathrm{\big(1}-\gamma \big)\big)\big(N-\mathrm{1}\mathrm{\big)}}{\big(M-\mathrm{1}\mathrm{\big)}{\pi}_g}$ in eqn 11 (see the Appendix S2 for sample code and implementation in the phylosamp package).

### Correcting for overdispersion

In real disease systems, the number of people each infected individual infects is known to be imperfectly Poisson distributed. That is, the presence of “super-spreading events” leads to overdispersion in the transmission process.

To account for this, instead of assuming ${E}_{gg}\sim Pois\left({\lambda}_g\right)$ as specified in eqn 1, which assumes the mean and variance of the distribution are equivalent, we can instead allow ${E}_{gg}$ to follow a negative binomial distribution with an overdispersion parameter *k*. ${E}_{gg}$ following a negative binomial distribution will change our variance from eqn 5 to the following by the delta method:


(12) \begin{equation*} \mathit{\operatorname{var}}(U)={\sigma}_U^2=\frac{\big(N-\mathrm{1}\mathrm{\big)}}{R_A{\pi}_A^2M\big(M-\mathrm{1}\mathrm{\big)}}+\frac{\big(N-\mathrm{1}\mathrm{\big)}}{R_B{\pi}_B^2M\big(M-\mathrm{1}\mathrm{\big)}}+\frac{2}{k}. \end{equation*}

This updates the power calculation as follows:


(13) \begin{equation*} {P}_{overdisp}=\mathrm{1}-\varPhi \left[{z}_{1-\alpha }-\frac{\log \left(\frac{R_B}{R_A}\right)}{\sqrt{\left[1+\frac{R_A}{d^2{R}_B}\right]\frac{\big(N-\mathrm{1}\mathrm{\big)}}{R_A{\pi}_A^2M\big(M-\mathrm{1}\mathrm{\big)}}+\frac{2}{k}}}\right]. \end{equation*}

Therefore, the total sample size needed to achieve $1-\beta$ power under ${H}_1:{R}_B/{R}_A>1$ given the overdispersion parameter, *k* is


(14) \begin{equation*} M\!=\!\frac{1}{2}\!\left[\!1\!+\!\sqrt{\!4\!\left[\!1\!+\!\frac{R_A}{R_B{d}^2}\!\right]\!\frac{1}{\log\! \big({R}_B/{R}_A{\!\big)}^2/ \big({z}_{1-\alpha }+{z}_{1-\beta }\!{\big)}^2\!-\!\mathrm{2}/k}\frac{\big(N-\mathrm{1}\mathrm{\big)}}{R_A{\pi}_A^2}\!+\!1}\!\right]\!. \end{equation*}

For operating characteristics of this estimator, see Figures S3 and S4 in Appendix S2.

### Two-sided hypothesis tests

We can extend this last equation to the 2-sided scenario where ${H}_0:{R}_A={R}_B$ and ${H}_1:{R}_B/{R}_A\ne 1$ as follows, where the power for the 2-sided test is


(15) \begin{equation*} {P}_{twosided}=\mathrm{1}-\varPhi \left[{z}_{1-\alpha /\mathrm{2}}-\frac{\left|\log \left(\frac{R_B}{R_A}\right)\right|}{\sqrt{\left[1+\frac{R_A}{d^2{R}_B}\right]\frac{\big(N-\mathrm{1}\mathrm{\big)}}{R_A{\pi}_A^2M\big(M-\mathrm{1}\mathrm{\big)}}}}\right]. \end{equation*}

Therefore, the total sample size needed to achieve $1-\beta$ power under ${H}_1:{R}_B/{R}_A\ne 1$ is


(16) \begin{equation*} M=\frac{1}{2}\left[1+\sqrt{4\left[1+\frac{R_A}{R_B{d}^2}\right]\frac{\big({z}_{1-\alpha /\mathrm{2}}+{z}_{1-\beta }{\big)}^2}{\log \big({R}_B/{R}_A{\big)}^2}\frac{\big(N-\mathrm{1}\mathrm{\big)}}{R_A{\pi}_A^2}+1}\right]. \end{equation*}

### Sample size correction

The asymptotic properties of these methods have been shown to overestimate the needed sample size when there is imbalance between groups such that ${\pi}_B>{\pi}_A$ and underestimate when ${\pi}_B<{\pi}_A$_._^12^^,^^13^ To this end, we have implemented a simulation-based approach that provides a corrected sample size for a given desired power under specified parameters. This is implemented by searching through potential sample sizes for given input parameters using a binary search until a sample size is found that provides the requested nominal power. Functions to implement these simulations are available in the phylosamp R package, and sample code is provided in Appendix S2.^14^

## Validation methods

**Figure 2:**
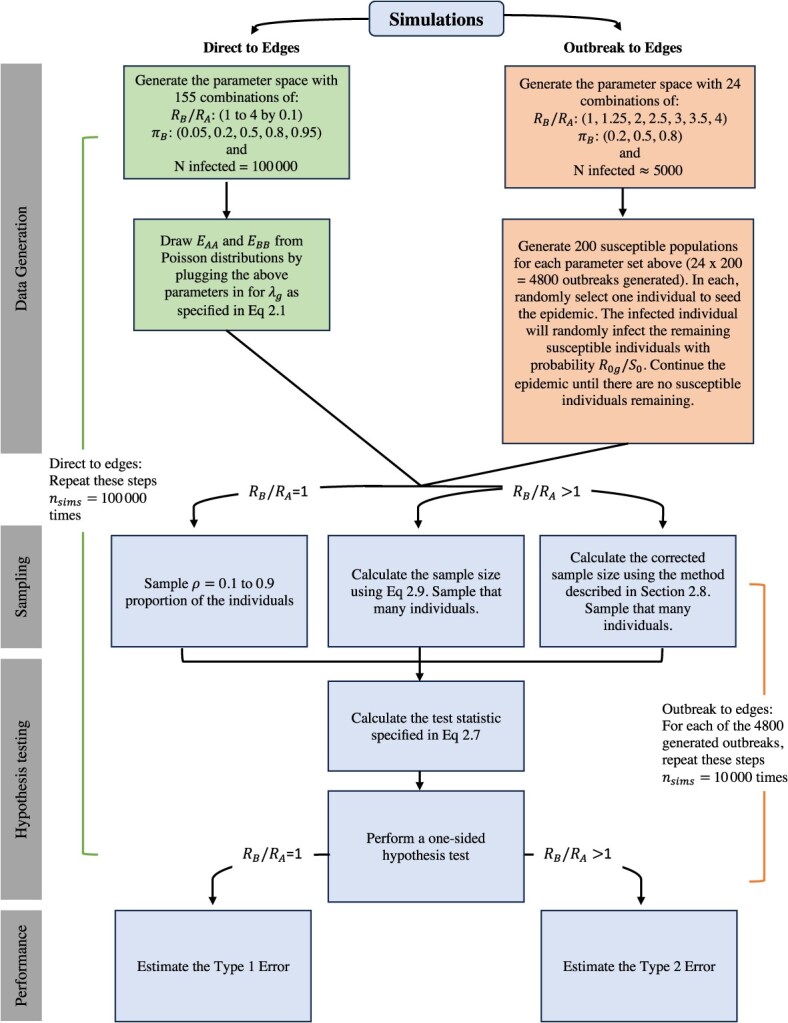
Schema describing the simulation process.

We validate our approach using 2 simulation frameworks. In the first, “direct to edges” scenario, we assume our distributional assumptions about observed edges are correct and explore a wide range of the parameter space. In the second, “outbreak to edges” scenario, we simulate outbreaks and then sample from them. In this latter approach, we explore a smaller range of the parameter space due to computational constraints. Figure 2 displays a schema describing the simulation process.

### Direct to edges simulations

In the direct to edges scenario, we draw the number of edges in each group directly from Poisson distributions using the following data generating mechanisms. With the goal of covering a reasonable range of transmission differences and group imbalance, we examine 31 values for the ratio ${R}_B/{R}_A$ as follows from 1 to 4 by 0.1 and 5 values for the proportion of the population in the numerator, ${\pi}_B=0.$05, 0.2, 0.5, 0.95, resulting in 155 scenarios. We simulate 100 000 “epidemics” under each scenario. All epidemics have *N* = 100 000 infected individuals (a large number was selected to ensure achievable sample sizes). In each generated epidemic, we simulated the total number of like edges in each group, ${E}_{AA}$and ${E}_{BB}$, by sampling from a Poisson distribution with ${\lambda}_g$, as specified in eqn 1 based on the specifications of each scenario. We perform a 1-sided hypothesis test examining the alternative hypothesis, ${H}_1:{R}_B/{R}_A>1$. For scenarios where ${R}_B/{R}_A>1$. *M* is specified using the eqn 9, setting the nominal power to 80% ($\beta =\mathrm{0.2}$) and $\alpha =\mathrm{0.05}$. Additionally, we calculate a “corrected” sample size, ${M}_{corrected}$ using a simulation based approach. For scenarios where ${R}_B/{R}_A=1$_,_ we sample the $\rho =0.1$ to 0.9 proportion of the epidemic. We then use the generated ${E}_{AA}$and ${E}_{BB}$values to calculate the test statistic specified in eqn 7 to perform the 1-sided hypothesis test in order to estimate the type 1 (when ${R}_B/{R}_A=1$) and type 2 (otherwise) errors.

Additionally, we consider scenarios where there is imperfect sensitivity and specificity in our identification of transmission linked case pairs. We examine sensitivity levels of 0.5, 0.75, 0.9, and 0.99 and specificity levels of 0.99, 0.999, and 0.9999.

### Outbreak to edges simulations

#### Generating the outbreaks

We consider a wide range of potential ratios of ${R}_B/{R}_A$ (1, 1.25, 1.5, 2, 2.5, 3, 3.5, 4) and 3 values for the proportion of the population in the numerator, ${\pi}_B=0.2$, 0.5, 0.8, resulting in 24 scenarios. We simulate 200 outbreaks under each scenario. In each generated outbreak, we simulate the total number of susceptible individuals, ${S}_0$ from a Poisson distribution with the mean, ${\lambda}_S$, selected such that each scenario would generate outbreaks of size 5 000 on average. Each individual was assigned a group, *A* or *B*, with probability ${\pi}_A$ and ${\pi}_B$ as specified. Simulated individuals moved through 3 stages: S (susceptible), I (infectious), R (recovered). We randomly selected 1 individual to seed the epidemic. The infected individual infected remaining susceptible individuals with probability ${p}_g={R}_{0g}/{S}_0$, where ${R}_{0g}$ is the basic reproductive number for group $G=g$. Infected individuals were infectious for 1 time step (ie, the epidemic progresses in discrete generations). The epidemic continued until there were no susceptible individuals remaining.

#### Sampling from the outbreaks

We performed a 1-sided hypothesis test examining the alternative hypothesis ${H}_1:{R}_B/{R}_A>$1. For scenarios where ${R}_B/{R}_A>$1, we used eqn 15 to calculate the sample size, setting the nominal power to 80% ($\beta =0.2$) and $\alpha =0.05.$ Additionally, we calculated a “corrected” sample size using a simulation based approach. For scenarios where ${R}_B/{R}_A=$1 sample $\rho =0.1$ to 0.9 proportion of the outbreak. Using the specified sample size, *M* (and additionally *M_corrected_* for the scenarios where ${R}_B/{R}_A$  $>1$) for each scenario, we randomly sampled 10 000 times for each outbreak and calculated the test statistic specified in eqn 7 in order to estimate the type 1 and type 2 errors.

## Results

Over multiple simulation studies we found that the derived test statistics and sample size calculations have the expected performance properties.

**Figure 3:**
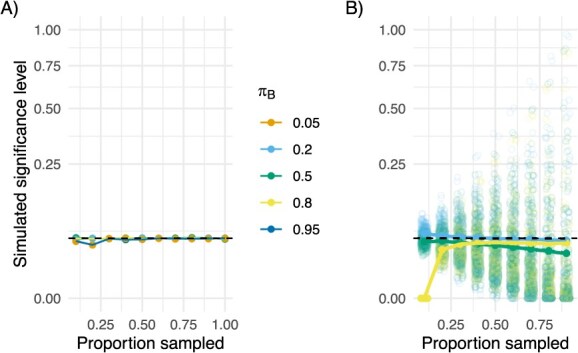
Simulated type 1 error ($\alpha =0.05$, shown in the dashed black line) varying the sampling fraction, $\rho$from 0 to 0.9 (x-axis) ${\pi}_B=0.05$ orange, only in the direct to edges simulation), 0.2 (light blue), 0.5 (green), 0.8 (yellow), 0.95 (dark blue, only in the direct to edges simulation). A, Observed type 1 error in the direct to edges simulation. B, Observed type 1 error in outbreak to edges simulation.

When there is no difference in transmissibility between groups, our approach successfully bounds the probability of erroneously detecting a significant difference. That is, the test statistic presented in eqn 7 has the expected type 1 error rate (or less in cases where a small proportion of the population is sampled) (Figure 3).

**Figure 4:**
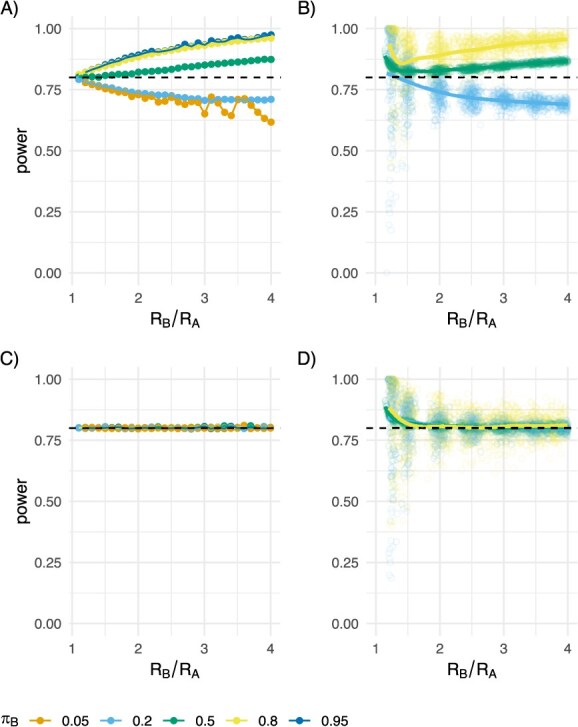
Simulated average power ($\alpha =0.05,\beta =0.2$), the nominal 80% power is shown with the dashed black line) varying the ratio of R values from 1 to 4 (x-axis) and ${\pi}_B=0.05$ (orange), 0.2 (light blue), 0.5 (green), 0.8 (yellow), 0.95 (dark blue). A, The direct to edges simulation. B, The outbreak to edges simulation, C, The direct to edges simulation with the “correction factor” applied. D, The outbreak to edges simulation with the “correction factor” applied.

Selecting sample sizes based on our approach successfully identified significant differences in transmissibility with the specified probability. In the setting of perfect detection of linked pairs, selecting sample sizes as per eqn 9 yielded the expected power in both direct to edges and outbreak to edges simulations (Figure 4A,B). That is, the actual power achieved is close to the target of 80% when the ratio of ${R}_B/{R}_A$is near 1. And it deviates as expected for the ratio of Poisson rates as per prior work by Ng and Tang (2005), with the target power being (roughly) achieved when equal proportions of the population are in each group, the power being higher than targeted when the more transmissible group is the majority and lower than targeted when the less transmissible group is the majority. We found that our simulation-based approach to correcting the sample size yields a study population that achieves the desired power in all cases (Figure 4C,D).

**Figure 5:**
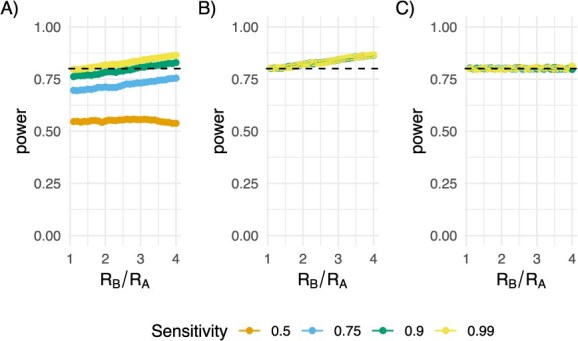
Simulated average power ($\alpha =0.05,\beta =0.2$, the nominal 80% power is shown in the dashed black line) varying the ratio of R values from 1 to 4 (x-axis). Here, the total number of infected individuals is 100 000, and the 2 groups are of equal size. Each line shows a different sensitivity: 0.5 (orange), 0.75 (light blue), 0.9 (green), 0.99 (yellow). A, This panel shows the impact on power if the sensitivity is not taken into account in the sample size calculation. B, This panel shows the impact on power when the sensitivity is taken into account in the sample size calculation, demonstrating that the original power is recovered. C, This panel shows the impact on power when the sensitivity is taken into account and the “correction factor” is applied, bringing the power back to 80% as specified.

We examined the impact of imperfect detection of transmission-linked pairs (ie, reduced sensitivity and specificity) on the power calculations. Sensitivity is the probability that we will detect a transmission-linked pair that is in our sample. When this is less than 1, we will need a larger sample size than indicated by eqn 9 (as confirmed by simulations, Figure 5). Equation 10 gives a corrected sample size (Figure 5B), and this can be further optimized using our simulation-based approach (Figure 5C).

**Figure 6:**
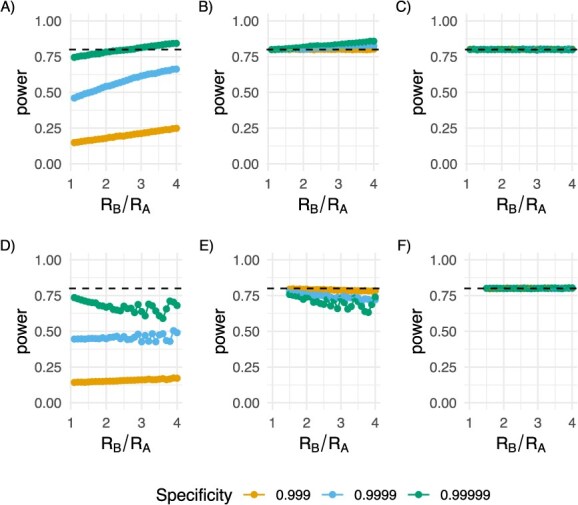
Simulated average power ($\alpha =0.05,\beta =0.2$, the nominal 80% power is shown in the dashed black line) varying the ratio of R values from 1 to 4 (x-axis). Here, the total number of infected individuals is 100 000. In the top 3 panels (A, B, C), the 2 groups are of equal size; in the bottom panels (D, E, F), 0, meaning 5% of the infected population, are from group B, and 95% are from group A. Each line shows a different specificity: 0.999 (orange), 0.9999 (blue), 0.99999 (green) A, This panel shows the impact on power if the specificity is not taken into account in the sample size calculation. B. This panel shows the impact on power when the specificity is taken into account in the sample size calculation, demonstrating that the specified power is recovered, although slightly overpowered, as is the case in general as the ratio of R values increases. C, This panel shows the impact on power when the specificity is taken into account in the sample size calculation and the “correction factor” is applied, demonstrating that the specified power is recovered, regardless of the ratio of R values. D, This panel shows the impact on power if the specificity is not taken into account in the sample size calculation. E, This panel shows the impact on power when the specificity is taken into account in the sample size calculation, demonstrating that the original power is not quite recovered, as is the case when the groups are imbalanced with the smaller group in the numerator (group B in this case). F, This panel shows the impact on power when the specificity is taken into account in the sample size calculation and the “correction factor” is applied, demonstrating that the specified power is recovered, regardless of the ratio of R values.

Specificity is the probability that we will not link 2 people in our sample who are in fact not a transmission-linked pair. Perhaps more intuitively, 1-specificity is the probability that 2 random people in our sample would be considered a transmission-linked pair if they are in fact not linked. Because imperfect specificity biases estimates towards the null (if errors are independent of the characteristic of interest), imperfect specificity increases the needed sample size beyond that indicated in eqn 15 (Figure 6A). This corrected by using an iterative solving of eqn 15 (Figure 6B) and further optimized by using our simulation based approach (Figure 6C).

For examples of sample sizes given in specific parameters, see Figures S1 and S2 in Appendix S2. For examples of the impact of overdispersion on the presented operating characteristics, see Figures S3 and S4 in Appendix S2.

## Discussion

Here, we have presented a simple estimator for the ratio of reproductive numbers between 2 groups based on the identification of transmission-linked pairs and provided validated tools for sample size calculation based on this estimator. We have provided extensions to our method to correct for imperfect identification of linked pairs, overdispersion in the transmission process (ie, the presence of superspreading), and group imbalance. In doing so, we aimed to provide accessible methods for guiding study design, while retaining enough sophistication to deal with the imperfect nature of molecular inferences about transmission. In order to aid adoption, all methods are implemented in the phylosamp R package available on CRAN (https://github.com/HopkinsIDD/phylosamp). Use of this approach should lead to more efficient study designs and better evaluation of information gleaned from molecular studies of transmission, though more work is needed to address situations with more complex dynamics (e.g., where transmission is assortative within groups).

Because our approach is based only on linkage among individuals within the same group, it is likely that it provides a conservative estimate of the amount of information available in a given sample. If techniques or data are available that allow us to leverage the information provided by transmission pairs spanning groups, the sample size rendered by our approach should be more than adequate to test the hypothesis of differential transmission between groups (i.e., it should represent an upper limit on the required sample size). For example, if the direction of transmission could be known or inferred, linkages between individuals with different characteristics could be better utilized. Likewise, sophisticated techniques that take advantage of phylogenetic relationships beyond direct linkage to characterize transmission pathways may be able to more efficiently estimate the ratio of reproductive numbers between groups, particularly if characteristics represent distinct populations (e.g., different geographic regions), thereby reducing the needed sample size. While more work is needed to make studies with such additional information maximally efficient, use of our method should ensure that they would be adequately powered.

Currently, our method assumes that the ratio of empirical reproductive numbers between groups is constant over the time period of the data collected. While this assumption is often reasonable, it is possible that this could be violated, for example, if the behaviors of a certain group differentially change compared to the other group over a period of time. This could be handled by adjusting for the potential factor that influences the differential behavior; a natural extension of this work could be the ability to adjust the estimator and sample size calculation in the presence of covariate adjustment, both for confounding control and to potentially increase the precision of the estimate. We hope to address this in future work.

Our assumption of transmission being independent of the relationship between infector and infectee characteristics could be violated if there is assortative mixing or if particular kinds of contacts were more likely to lead to transmission than others. For example, different sex acts have different probabilities of transmission for many STIs and are more likely to occur in certain types of partnerships than others. Extension of our approach to this setting is a clear avenue for future work.

A further limitation of this approach is the assumption that sequences come from a random sample of the infected population. This may be a particular challenge for molecular studies as clinical or other convenience samples are so often used. When the random sampling assumption is violated, our methods may still be valid or correctable using standard weighting techniques; doing so would require careful consideration of the sampling process and potential biases.

However, it should be noted that our approach is robust to differential susceptibility between groups. To understand why this is the case consider that, absent assortative mixing, when we are looking at a sample of only infected individuals we are unable to distinguish differences in susceptibility between groups from differences in the proportion of individuals in each group in the underlying population (i.e., the ratio of individuals from each group in the infected population is simply modified by the ratio of their susceptibility). Since our estimator corrects for these proportions, it is not biased by differential susceptibility between these populations. However, this also means that it is impossible to distinguish differential susceptibility between groups from differences in their prevalence in the source population without information on uninfected individuals, regardless of the sophistication of the techniques used.

As molecular data become increasingly important in the practice of infectious disease epidemiology, it is important that we have accessible and robust techniques to guide the collection of samples for a wide variety of inferential goals. The approaches presented here are one small step on the path to creating this set of tools. Though these methods are limited to examining differential transmission between people with different characteristics absent assortitivity, such focused analyses are the key building blocks on the road to developing more sophisticated techniques.

## Supplementary material

Supplementary material is available at the *American Journal of Epidemiology* online.

## Funding

None declared.

## Conflict of interest

The authors declare no conflicts of interest.

## Supplementary Material

Web_Material_kwae378
