# Supplementary material for: Power and sample size calculations for testing the ratio of reproductive values in phylogenetic samples
Source: Am J Epidemiol. 2024 Oct 10;194(8):2367–75. doi: 10.1093/aje/kwae378 (PMC12342956; doi:10.1093/aje/kwae378)
Supplement: Web_Material_kwae378 [file web_material_kwae378.zip › appendix.pdf]

# Supplementary Material: Power and sample size calculations for testing the ratio of reproductive values in phylogenetic samples

Lucy D'Agostino McGowan Shirlee Wohl Justin Lessler

## Table of contents

**Appendix S1** **1**
Distributional Assumptions . . . . . . . . . . 1
Sample size derivation . . . . . . . . . . 3

**Appendix S2** **4**
Sample code . . . . . . . . . . 4
Estimated sample sizes . . . . . . . . . . 6
Overdispersion . . . . . . . . . . 6

**References** **7**

## Appendix S1

### Distributional Assumptions

Below is intuition behind our derivations. Let's begin by assuming we are observing the whole outbreak of size $N$. A transmission pair is defined as two individuals $i$ and $j$ who are connected by an edge. Assume $e_{ij}$ is the probability that individual $i$ infected individual $j$. The total number of people infected by individual $i$ is the sum, which we assume is Poisson distributed $e_{i\cdot} \sim Pois(R_{g_i})$, where $R_{g_i}$ is the empirical reproductive number for individual $i$ who has group level $G = g$ (1).

The total number of edges is equal to:

$$E = \sum_{i=1}^{N} e_{i.}$$

The sum of $N$ Poisson distributed random variables is Poisson, hence we can define the total number of edges in an outbreak as:

$$E \sim Pois(NR_{pop})$$

Assuming there are two groups $g \in \{A, B\}$, the weighted average of $R_g$ across both groups, also known as $R_{pop}$, can be written as $\pi_A R_A + \pi_B R_B$, where $\pi_g$ is the proportion of the outbreak belonging to group $G = g$.

The total number of edges where a member of group $G = g$ was the infector can be written as:

$$E_{g\cdot} = \sum_{\{i:G_i=g\}} e_{i\cdot}$$

The total number of individuals for whom $g_i = g$ is $N\pi_g$, therefore $E_{g\cdot} \sim Pois(N\pi_g R_g)$.

The total number of individuals in group $G = g$ who are infected by individual $i$ is the sum of $e_{ij}$ where $G_j = g$ is:

$$e_{i\cdot_{\{G_j=g\}}} = \sum_{j:G_j=g} e_{ij} \sim Pois(\pi_{g_j} R_{g_i})$$

Since the proportion of the susceptible population belonging to group $G = g$, that is the group of the infectee, is $\pi_{g_j}$.

The total number of edges where the infector and infectee are both members of the same group, $G = g$ is as follows:

$$E_{gg} = \sum_{i:G_i=g} e_{i\cdot_{\{G_j=g\}}}$$

The total number of individuals for whom $G_i = g$ in an outbreak of size $N$ is $N\pi_g$, therefore $E_{gg} \sim Pois(N\pi_g^2 R_g)$.

The above intuition assumes we are observing the entire outbreak. Under a simple random sample, where $M/N = \rho$ proportion of the outbreak are sampled, the probability of sampling infector $i$ is $M/N$ and the probability of sampling infector $i$'s infectee $j$ is $\frac{M-1}{N-1}$. Therefore, the expected number of total number of edges in a sample of size $M$ from an outbreak of size $N$ is:

$$\mathbb{E}[E] = \frac{M(M-1)}{N-1} R_{pop}$$

where $E \sim Pois\left(\frac{M(M-1)}{N-1} R_{pop}\right)$. Similarly, the expected number of like edges, $E_{gg}$ in a sample is:

$$\mathbb{E}[E_{gg}] = \frac{M(M-1)}{N-1} \pi_g^2 R_g$$

where $E_{gg} \sim Pois\left(\frac{M(M-1)}{N-1} \pi_g^2 R_g\right)$.

## Sample size derivation

$$-z_{1-\beta} = z_{1-\alpha} - \frac{\log\left(\frac{R_B}{R_A}\right)}{\sqrt{\left[1 + \frac{R_A}{d^2 R_B}\right] \frac{(N-1)}{R_A \pi_A^2 M(M-1)}}}$$

$$\sqrt{\left[1 + \frac{R_A}{d^2 R_B}\right] \frac{(N-1)}{R_A \pi_A^2 M(M-1)}} = \frac{\log\left(\frac{R_B}{R_A}\right)}{z_{1-\alpha} + z_{1-\beta}}$$

$$\left[1 + \frac{R_A}{d^2 R_B}\right] \frac{(N-1)}{R_A \pi_A^2 M(M-1)} = \frac{\log\left(\frac{R_B}{R_A}\right)^2}{(z_{1-\alpha} + z_{1-\beta})^2}$$

$$M(M-1) = \left[1 + \frac{R_A}{d^2 R_B}\right] \frac{(N-1)}{R_A \pi_A^2} \frac{(z_{1-\alpha} + z_{1-\beta})^2}{\log\left(\frac{R_B}{R_A}\right)^2}$$

$$M^2 - M + \frac{1}{4} = \left[1 + \frac{R_A}{d^2 R_B}\right] \frac{(N-1)}{R_A \pi_A^2} \frac{(z_{1-\alpha} + z_{1-\beta})^2}{\log\left(\frac{R_B}{R_A}\right)^2} + \frac{1}{4}$$

$$\left(M - \frac{1}{2}\right)^2 = \left[1 + \frac{R_A}{d^2 R_B}\right] \frac{(N-1)}{R_A \pi_A^2} \frac{(z_{1-\alpha} + z_{1-\beta})^2}{\log\left(\frac{R_B}{R_A}\right)^2} + \frac{1}{4}$$

$$M - \frac{1}{2} = \sqrt{\left[1 + \frac{R_A}{d^2 R_B}\right] \frac{(N-1)}{R_A \pi_A^2} \frac{(z_{1-\alpha} + z_{1-\beta})^2}{\log\left(\frac{R_B}{R_A}\right)^2} + \frac{1}{4}}$$

$$M = \sqrt{\left[1 + \frac{R_A}{d^2 R_B}\right] \frac{(N-1)}{R_A \pi_A^2} \frac{(z_{1-\alpha} + z_{1-\beta})^2}{\log\left(\frac{R_B}{R_A}\right)^2} + \frac{1}{4}} + \frac{1}{2}$$

$$M = \frac{1}{2}\left[\sqrt{4\left[1 + \frac{R_A}{d^2 R_B}\right] \frac{(N-1)}{R_A \pi_A^2} \frac{(z_{1-\alpha} + z_{1-\beta})^2}{\log\left(\frac{R_B}{R_A}\right)^2} + 1} + 1\right]$$

# Appendix S2

## Sample code

Below is code to estimate the sample size needed to estimate the ratio between $R_A : 0.83$, $R_B : 1.67$, where the prevalence of the characteristic of interest in the infected population is 0.8 for group A (and therefore 0.2 for group B). We are targeting 80% power for a one-sided hypothesis test (testing $H_0 : R_B/R_A = 1$ vs $H_1 : R_B/R_A > 1$) with a significance level of $\alpha = 0.05$. The full outbreak size is 5,000.

```
library(phylosamp)
set.seed(7)
relR_samplesize(R_a = 0.83,
                R_b = 1.67,
                p_a = 0.8,
                N = 5000,
                alpha = 0.05,
                power = 0.8,
                alternative = "greater")
```

This estimates that we need a sample size of `1033` given the above parameters. If instead of a one-sided hypothesis test we wanted to conduct a two sided hypothesis test, we would run the following.

```
relR_samplesize(R_a = 0.83,
                R_b = 1.67,
                p_a = 0.8,
                N = 5000,
                alpha = 0.05,
                power = 0.8,
                alternative = "two_sided")
```

A two-sided test with the above parameters is estimated to require a sample size of `1164`. Since we have imbalanced groups (80% are in group A vs 20% in group B) we know that this will be an underestimate for the sample size needed, assuming that our distributional assumptions are met. We can correct for this by using our simulation-based approach by setting the parameter `correct_for_imbalance = TRUE` as follows.

```
relR_samplesize(R_a = 0.83,
                R_b = 1.67,
                p_a = 0.8,
```

```
                N = 5000,
                alpha = 0.05,
                power = 0.8,
                alternative = "two_sided",
                correct_for_imbalance = TRUE)
```

After correcting for the group imbalance using our simulation approach, a two-sided test with the above parameters is estimated to require a sample size of `1273`. Let's assume that we expect to imperfectly observe linkage pairs such that the sensitivity is 0.9 and the specificity is 0.999.

```
relR_samplesize(R_a = 0.83,
                R_b = 1.67,
                p_a = 0.8,
                N = 5000,
                alpha = 0.05,
                power = 0.8,
                alternative = "two_sided",
                sensitivity = 0.9,
                specificity = 0.999,
                correct_for_imbalance = TRUE)
```

After incorporating sensitivity and specificity, our required sample size increases to `2540`. Let's assume that there is overdispersion present, with an overdispersion parameter of `k = 35`. This overdispersion parameter implies that you are assuming a negative binomial distribution as opposed to a Poisson distribution, as is the default. An overdispersion parameter of infinity would be equivalent to the default.

```
relR_samplesize(R_a = 0.83,
                R_b = 1.67,
                p_a = 0.8,
                N = 5000,
                alpha = 0.05,
                power = 0.8,
                alternative = "two_sided",
                correct_for_imbalance = TRUE,
                overdispersion = 35)
```

After allowing for overdispersion, a two-sided test with the above parameters is estimated to require a sample size of `4434`.

## Estimated sample sizes

Figure S1 and Figure S2 demonstrate the estimated sample sizes needed given specific parameters.

## Overdispersion

Figure S3 and Figure S4 show the type 1 error and power in the presence of overdispersion. The type 1 error is well controlled despite overdispersion; overdispersion tends lead to increased power.

# References

1. Lloyd-Smith JO, Schreiber SJ, Kopp PE, et al. Superspreading and the effect of individual variation on disease emergence. *Nature.* 2005;438(7066):355–359.

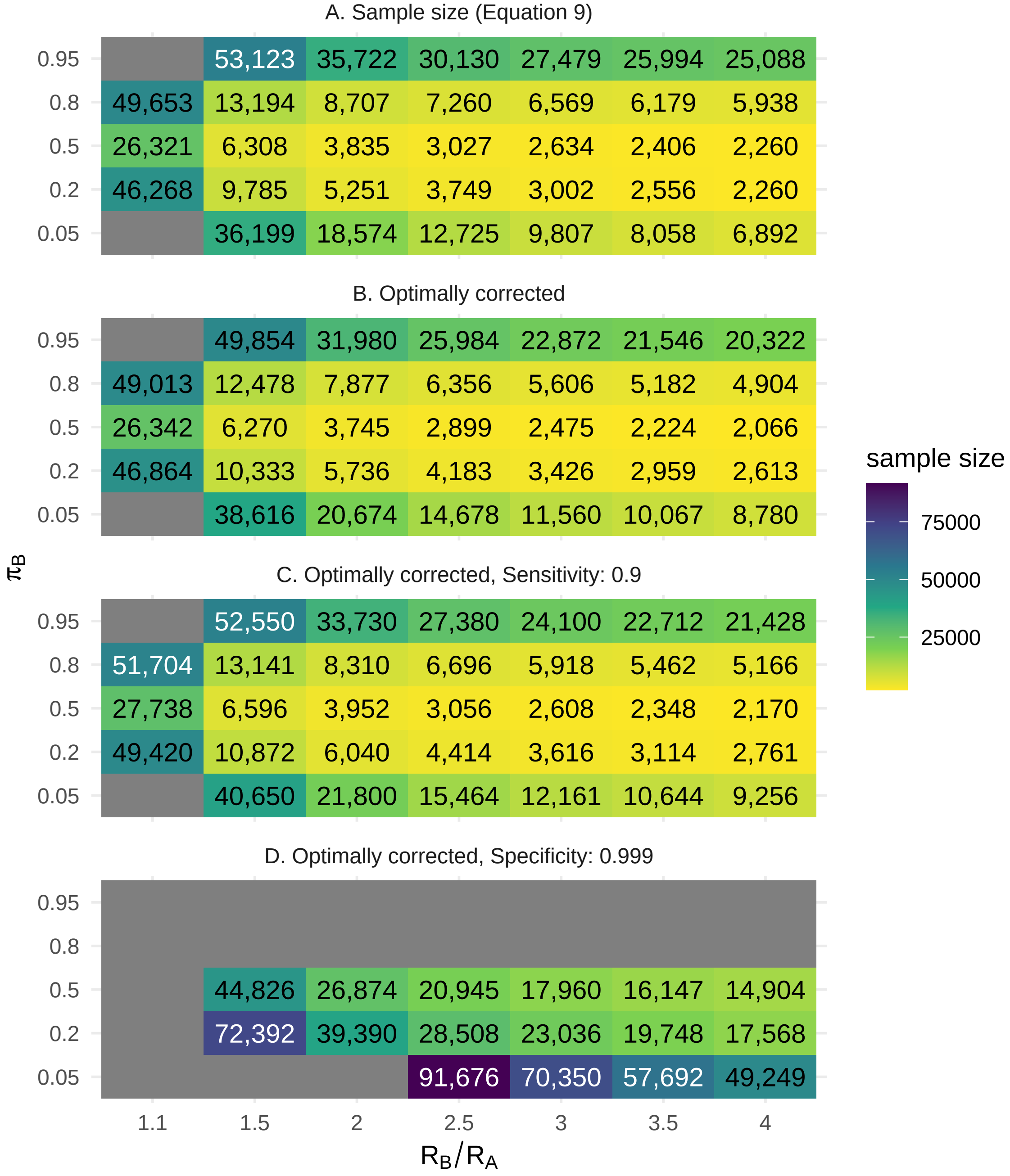


Figure S1: The sample size needed for an epidemic of size 100,000 with $\pi_B$ as specified on the y-axis and the ratio between $R_B$ and $R_A$ as specified on the y-axis, chosen such that $R_A\pi_A + R_B\pi_B = 1$ to achieve 80% power for a one-sided test where $\alpha = 0.05$. The top two panels (A, B) correspond to a perfectly observed sample (i.e. no sensitivity or specificity). Panel C has a sensitivity of 0.9, the bottom panel (D) has a specificity of 0.999. Panel A uses the sample size calculation given in Equation 9. Panels B-D use the simulation-based optimal sample size, using methods in the `phylosamp` R package. Grey boxes indicate parameter combinations for which the sample size needed to detect the requested change exceeds the size of the epidemic.

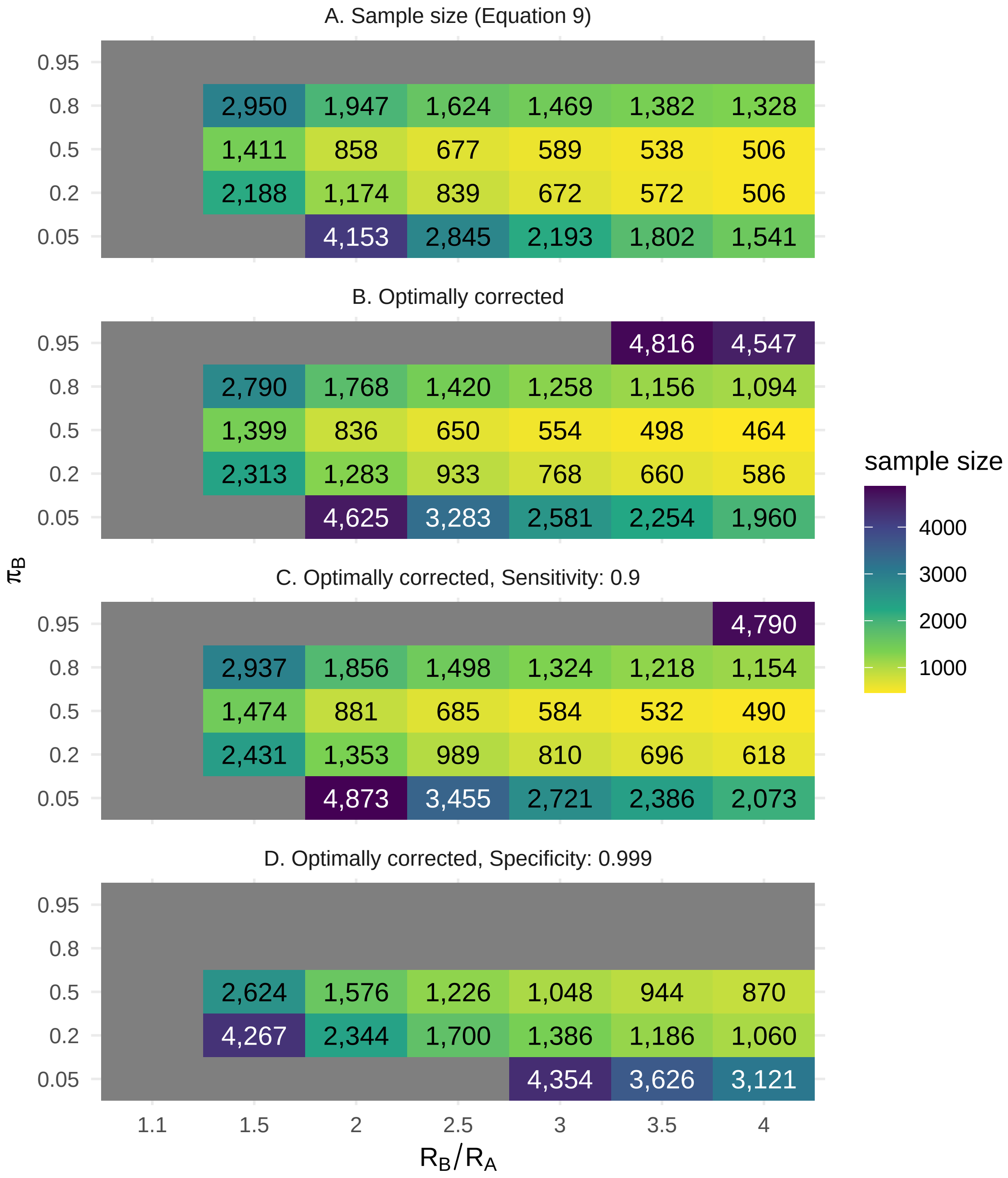


Figure S2: The sample size needed for an outbreak of size 5,000 with $\pi_B$ as specified on the y-axis and the ratio between $R_B$ and $R_A$ as specified on the y-axis, chosen such that $R_A\pi_A + R_B\pi_B = 1$ to achieve 80% power where $\alpha = 0.05$ . The top two panels (A, B) correspond to a perfectly observed sample (i.e. no sensitivity or specificity). Panel C has a sensitivity of 0.9, the bottom panel (D) has a specificity of 0.999. Panel A uses the sample size calculation given in Equation 9. Panels B-D use the simulation-based optimal sample size, using methods in the `phylosamp` R package. Grey boxes indicate parameter combinations for which the sample size needed to detect the requested change exceeds the size of the outbreak.

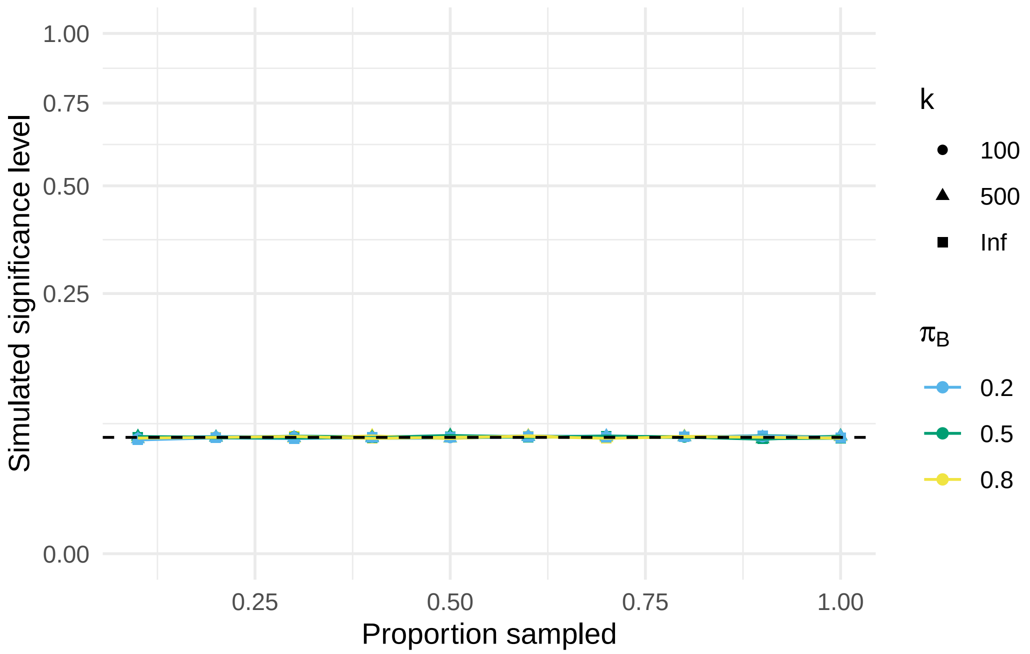


Figure S3: Simulated type 1 error ($\alpha = 0.05$, shown in the dashed black line) varying the sampling fraction, $\rho$, from 0 to 0.9 (x-axis) $\pi_B = 0.2$ (light blue), 0.5 (green), 0.8 (yellow), across three overdispersion parameters (k = 100, 500, Inf, where the infinite dispersion parameter is equivalent to the default Poisson assumption).

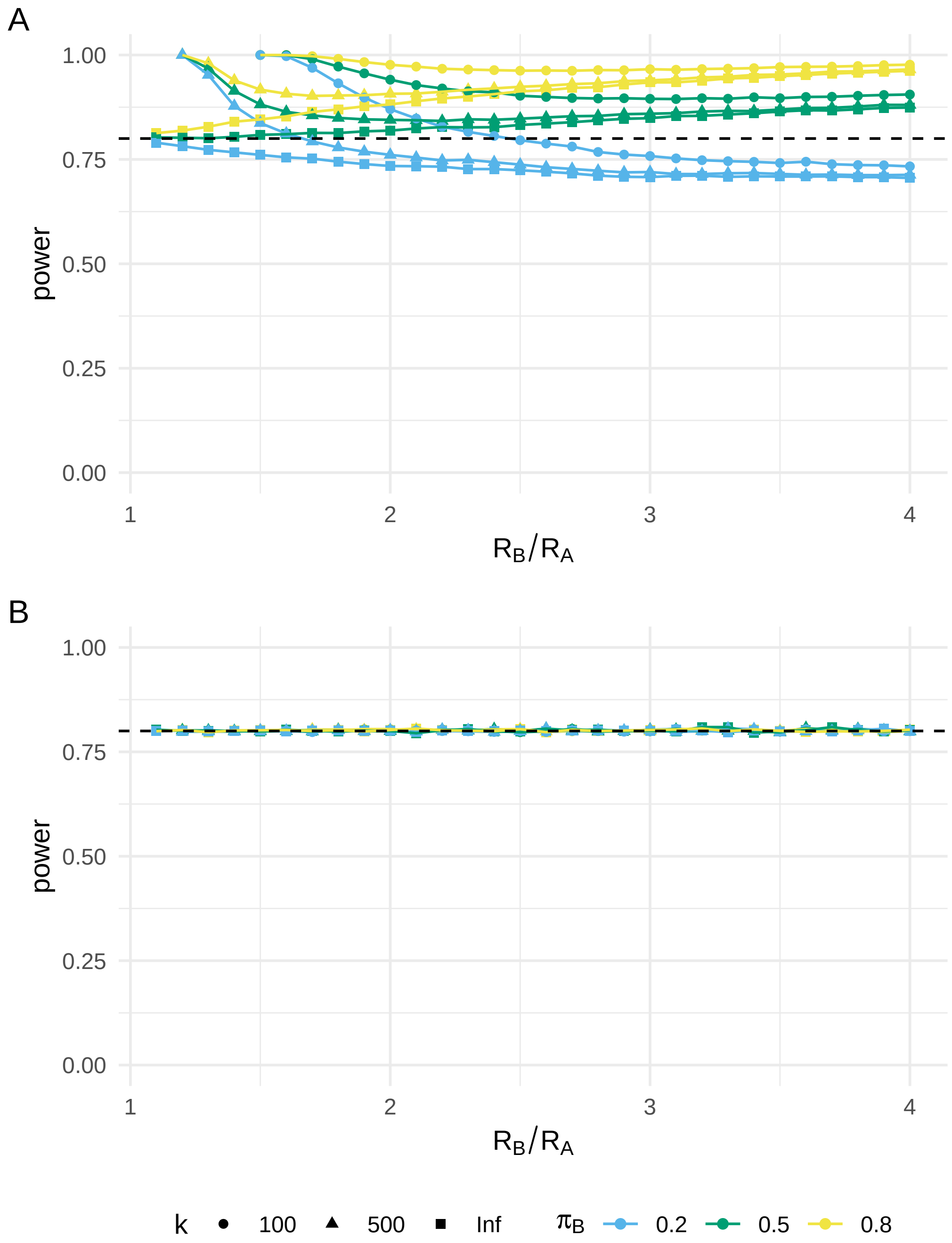


Figure S4: Simulated average power ($\alpha = 0.05, \beta = 0.2$, the nominal 80% power is shown with the dashed black line) varying the ratio of R values from 1 to 4 (x-axis) and $\pi_B = 0.2$ (light blue), 0.5 (green), 0.8 (yellow), across three overdispersion parameters (k = 100, 500, Inf, where the infinite dispersion parameter is equivalent to the default Poisson assumption). A. Shows the theoretical simulation, B. Shows the theoretical simulation with the 'correction factor' applied.
